# Novel *R*-roscovitine NO-donor hybrid compounds as potential pro-resolution of inflammation agents

**DOI:** 10.1016/j.bmc.2013.01.009

**Published:** 2013-04-01

**Authors:** Gabriele Montanaro, Massimo Bertinaria, Barbara Rolando, Roberta Fruttero, Christopher D. Lucas, David A. Dorward, Adriano G. Rossi, Ian L. Megson, Alberto Gasco

**Affiliations:** a Dipartimento di Scienza e Tecnologia del Farmaco, Università degli Studi di Torino, Via P. Giuria 9, 10125 Torino, Italy; b MRC Centre for Inflammation Research, Queen’s Medical Research Institute, University of Edinburgh Medical School, Edinburgh EH16 4TJ, United Kingdom; c Free Radical Research Facilities, Department of Diabetes and Cardiovascular Science, The Univeristy of the Highlands & Islands, Inverness IV2 3JH, United Kingdom

**Keywords:** *R*-Roscovitine, Multi-target drugs, Apoptosis, Nitric oxide donors

## Abstract

Neutrophils play a pivotal role in the pathophysiology of multiple human inflammatory diseases. Novel pharmacological strategies which drive neutrophils to undergo programmed cell death (apoptosis) have been shown to facilitate the resolution of inflammation. Both the cyclin-dependent kinase inhibitor (CDKi) *R*-roscovitine and nitric oxide (NO) have been shown to enhance apoptosis of neutrophils and possess pro-resolution of inflammation properties. In order to search for new multi-target pro-resolution derivatives, here we describe the design, synthesis and investigation of the biological potential of a small series of hybrid compounds obtained by conjugating *R*-roscovitine with two different NO-donor moieties (compounds **2**, **9a**, **9c**). The synthesized compounds were tested as potential pro-resolution agents, with their ability to promote human neutrophil apoptosis evaluated. Both compound **9a** and **9c** showed an increased pro-apoptotic activity when compared with either *R*-roscovitine or structurally related compounds devoid of the ability to release NO (*des*-NO analogues). Inhibition of either NO-synthase or soluble guanylate cyclase did not affect the induction of apoptosis by the *R*-roscovitine derivatives, similar to that reported for other classes of NO-donors. In contrast the NO scavenger PTIO prevented the enhanced apoptosis seen with compound **9a** over *R*-roscovitine. These data show that novel compounds such as CDKi–NO-donor hybrids may have additive pro-resolution of inflammation effects.

## Graphical abstract

NO-donor *R*-roscovitine derivatives were synthesized and evaluated in vitro as inflammation pro-resolving agents.

## 1 Introduction

Inflammation is the host response to tissue injury or infection, aimed at restoring damaged tissue to homeostasis. Following injury a variety of endogenous mediators are released which mediate the cardinal signs of inflammation: heat, redness, swelling, and pain. Vasoactive amines, lysozomal enzymes, cytokines, kinins, eicosanoids, clotting and fibrinolytic system products, nitric oxide (NO), and reactive oxygen species (ROS), are some of the important mediators involved. During an episode of acute inflammation a complex series of events occurs including the recruitment of leukocytes to the damaged tissue. Granulocytes (which comprise neutrophils, eosinophils and basophils) are the first leukocytes to be recruited, followed by monocyte/macrophage and lymphocyte infiltration into the inflamed tissue. The granulocytes are the body’s first line of innate immune defense and have the task of neutralizing the injurious stimulus.^1–3^ After removal of the inciting inflammatory stimulus it is essential that granulocytes die by apoptosis, a programmed and non-inflammatory form of cell death, in order that resolution of inflammation can occur. Apoptotic granulocytes are rapidly removed from inflammatory sites by macrophage phagocytosis with consequent dampening of inflammation.^4^ Previously it was believed that, after removal of the cause of the injury, the resolution of inflammation was merely a passive process secondary to the catabolism of pro-inflammatory mediators. More recently, an increasing number of studies have demonstrated that the resolution of inflammation is an active process involving several cellular processes including apoptosis and phagocytic clearance of apoptotic cells that can be modulated by endogenous specialized pro-resolving mediators (SPM). These include cyclopentanone prostaglandins, lipoxins, resolvins, protectins/neuroprotectins, and maresins.^3,5–7^ When inflammation resolution fails, the granulocytes can die by necrosis, an alternative form of cell death that involves rupture of cellular membrane with consequent release of granulocyte histotoxic contents into the extracellular milieu. This can perpetuate inflammation leading to chronicity, with dysregulated granulocyte apoptosis being involved in the pathogenesis of several human inflammatory diseases.^8^ The development of pharmacological agents or strategies capable of selectively increasing granulocyte apoptosis is a potential new therapeutic strategy to reduce both acute and chronic inflammation.^3,4^ The use of small molecule inhibitors of the serine/threonine cyclin-dependent kinases (CDKs) is one of the most promising strategies.^9^ There are at least 13 different CDK isoenzymes so far identified, and 25 different cyclins, the binding partners necessary for CDK activation. CDKs play essential functions in many biological processes including cell division, transcription, neuronal cell physiology, pain signaling, apoptosis, and RNA splicing.^10,11^ A variety of CDK inhibitor drugs have been described from different chemical classes including purines, pyrimidines, flavones, pyridopyrimidines, oxindoles, quinazolines and pyrazoles.^12,13^ *R*-Roscovitine **1**, a 2,6,9-trisubstituted purine, is one of the most studied. It is an inhibitor of CDK 1, 2, 5, 7 and 9 isoforms and has been widely investigated as a potential anticancer drug. The purine portion of the inhibitor binds to the adenine binding pocket of the enzyme, even if rotated with respect to the original ATP conformation. The crystal structure of the CDK2 and *R*-roscovitine complex is known, with the drug binding to the ATP binding pocket of the enzyme through hydrophobic and Van der Waals contacts, as well as hydrogen bonds between N(6) and N(7) of the purine ring and Leu83 of the enzyme. Another hydrogen bond involves O(1) of the aminobutanol lateral chain that binds to an area occupied by the ribose in the CDK2/ATP complex.^14^ Since *R*-roscovitine is able to enhance neutrophil apoptosis it also has a potential application as a pro-resolving anti-inflammatory agent.^9,15^ The ability of *R*-roscovitine to induce neutrophil apoptosis involves inhibition of CDKs, mainly CDK7 and CDK9, with consequent down-regulation of the pro-survival protein Mcl-1.^9,11^ NO also plays important roles in apoptosis, with it able to exert either pro-apoptotic or anti-apoptotic effects depending on the source and concentration of the NO and the cell type involved.^16^ A number of studies carried out with different NO-donors demonstrate the capacity of NO to induce apoptosis in neutrophils.^17–20^ In vivo evidence shows that supplementation of NO may be beneficial in inflammatory diseases with effects mediated by apoptosis of neutrophils.^21^ On this basis, we aimed to design, synthesise and investigate the biological potential of multi-target pro-resolution of inflammation derivatives based upon combined CDK inhibition and release of NO. To achieve this we designed new products in which *R*-roscovitine is linked through a hard bridge to moieties containing a NO-donor nitrooxy group (compound **9a**) or a NO-donor 3-furoxancarboxamide substructure (compound **9c**). Also compound **2** was considered in which *R*-roscovitine is linked to a nitrooxy substituted moiety through a vulnerable ester group. It is generally accepted that the NO-release from nitrooxy derivative occurs by enzymatic activation, whereas in the case of furoxan derivatives it occurs under the action of thiol co-factors.^22,23^ In this report the synthesis of these compounds and the results of a biological study aimed at exploring their ability to induce neutrophil apoptosis have been investigated. Also, compounds **9b**, **9d** which were structurally related to **9a**, **9c**, respectively, but devoid of the ability to release NO (*des*-NO analogues), were used for comparison.

## 2 Results and discussion

### 2.1 Chemistry

**Scheme 1:**
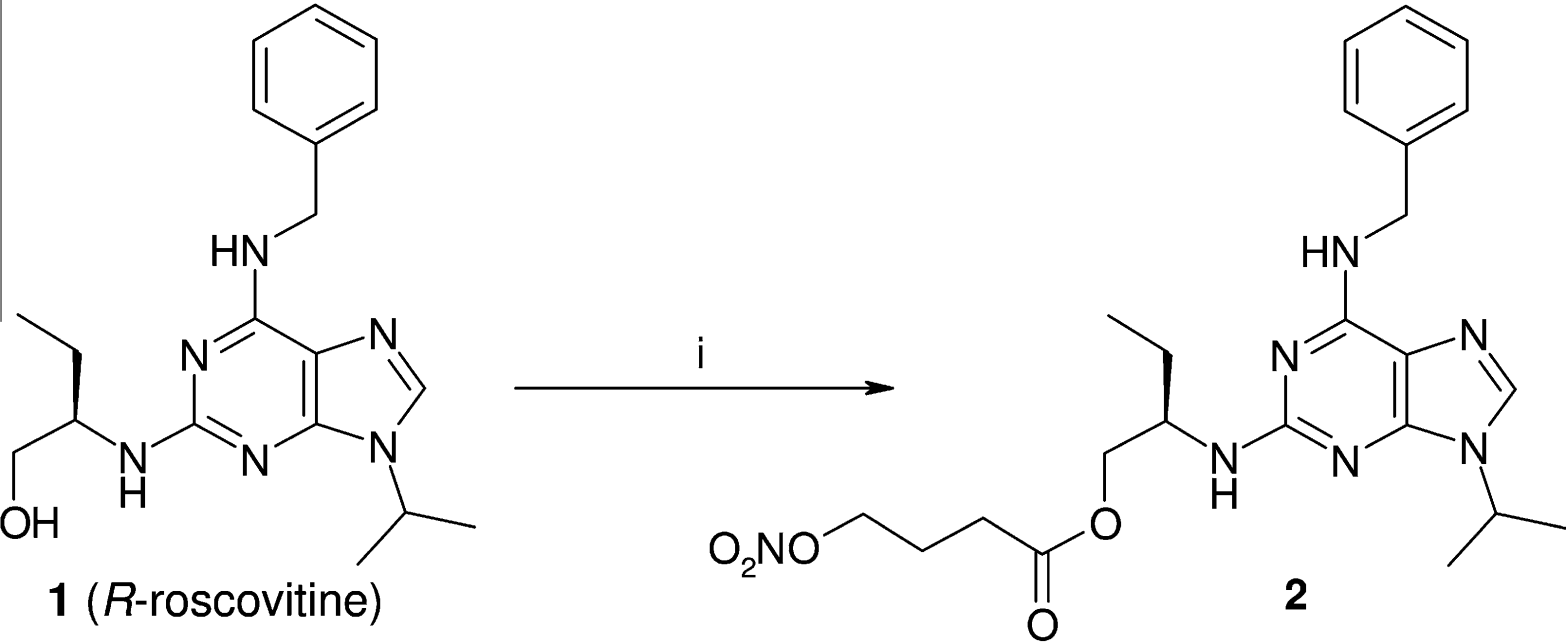
Preparation of derivative **2**. Reagents: (i) 4-nitrooxybutirric acid, EDC, DMAP, dry Pyr, 4 h, rt.

**Scheme 2:**
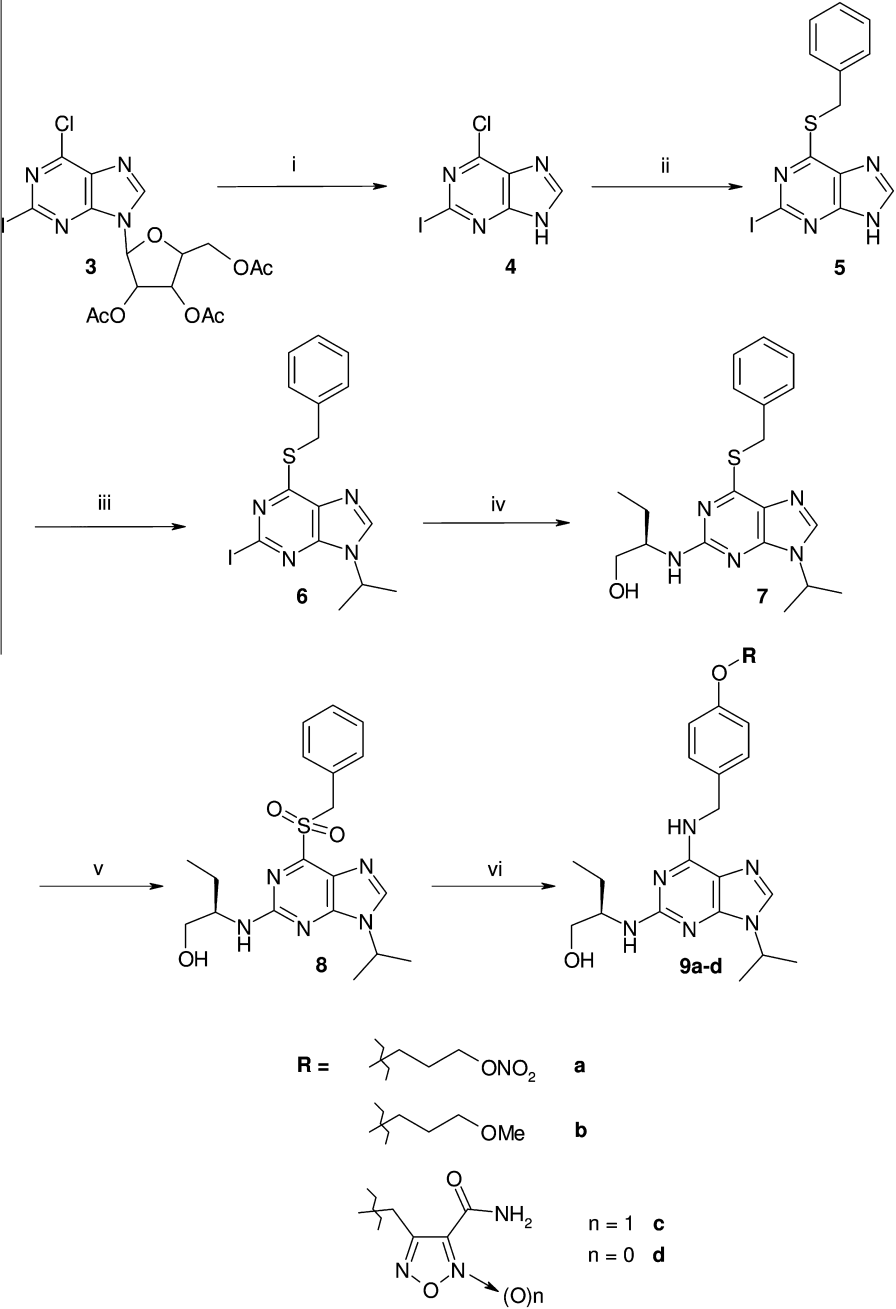
Preparation of derivatives **9a**–**d**. Reagents and conditions: (i) H_2_SO_4_, H_2_O/MeOH, 3 h, Δ; (ii) benzylmercaptane, DIPEA, EtOH, 4 h, Δ; (iii) 2-bromopropane, K_2_CO_3_, DMSO, 12 h, 50 °C; (iv) *R*-(2)-amino-1-butanol, *n*BuOH, 5 days, Δ; (v) *m*CPBA, CH_2_Cl_2_, 3 h, rt; (vi) **12**, **14**, **16** or **18**, Et_3_N, MeOH, 5 days, 40 °C.

**Scheme 3:**
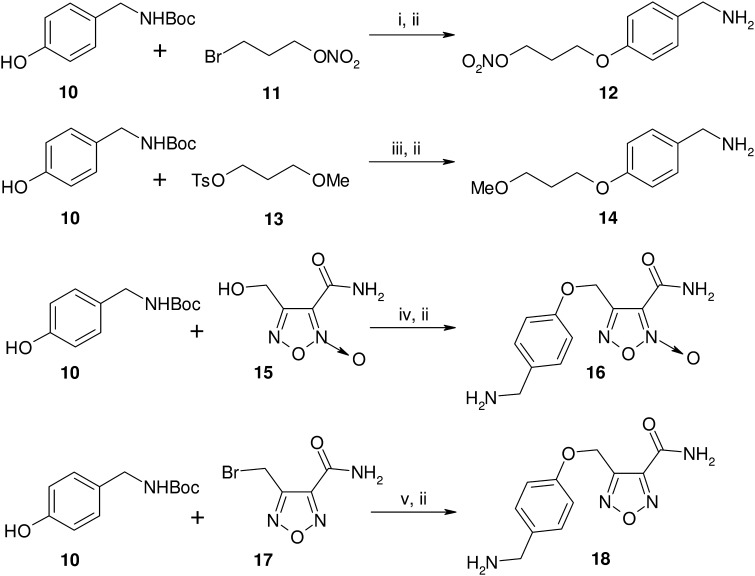
Preparation of amino derivatives **12**, **14**, **16**, **18**. Reagents and conditions: (i) K_2_CO_3_, KI, DMF, CH_3_CN, 20 h, 50 °C; (ii) TFA 10% CH_2_Cl_2_, 3 h, rt; (iii) *t*BuO^−^K^+^, dry THF, 4 h, rt → Δ; (iv) PPh_3_, DIAD, dry THF, 12 h, 0 °C → rt; (v) DBU, CH_2_Cl_2_, 12 h, rt.

The pro-drug **2** was easily obtained by coupling **1** with 4-nitrooxybutirric acid, under the action of 1-ethyl-3-(3-dimethylaminopropyl)carbodiimide (EDC), in the presence of catalytic amount of 4-dimethylaminopyridine (DMAP) in pyridine solution (Scheme 1). All the remaining products were synthesized starting from 6-benzylthio-2-iodo-9-isopropyl-9*H*-purine **6** (Scheme 2). This intermediate was already known in literature, but we prepared it through a partly modified route. The acetylated ribosyl group present in the guanosine derivative **3** was removed by the action of sulfuric acid to afford the already described 6-chloro-2-iodopurine **4**. Nucleophilic displacement of the chlorine with benzylmercaptane in the presence of diisopropylethylamine (DIPEA) led to the 2-iodo-6-benzylthio substituted purine **5**. The subsequent reaction with 2-bromopropane in DMSO in the presence of K_2_CO_3_ gave the N(9) regioselective alkylation, with resulting production of **6**. This intermediate, treated in refluxing *n*BuOH with pure *R*-(2)-amino-1-butanol, yielded the expected *R*-roscovitine thioanalogue **7**. This product was oxidized to the corresponding sulfonyl derivative **8** using *m*-chloroperbenzoic acid (*m*CPBA). Finally, **8** was treated with the appropriate benzyl amine derivative (**12**, **14**, **16**, **18**) in methanol solution in the presence of Et_3_N to give the target compounds **9a**–**d**. The preparation of the substituted benzylamines used in the reaction is outlined in Scheme 3. All the products were synthesized starting from *Boc*-protected *p*-hydroxybenzylamine **10**. Compounds **12**, **18**, were obtained following nucleophilic displacement by **10** of the bromine present in **11** and **17**, and subsequent *Boc*-deprotection with trifluoroacetic acid in CH_2_Cl_2_. In a similar manner **14** was prepared using the tosylate **13**. Finally **10**, treated with 4-hydroxymethyl furoxan-3-carboxyamide (**15**), in the presence of PPh_3_ and diisopropyl azodicarboxylate (DIAD) in THF solution (Mitsunobu procedure), afforded **16**.

### 2.2 Biological activity

**Figure 1:**
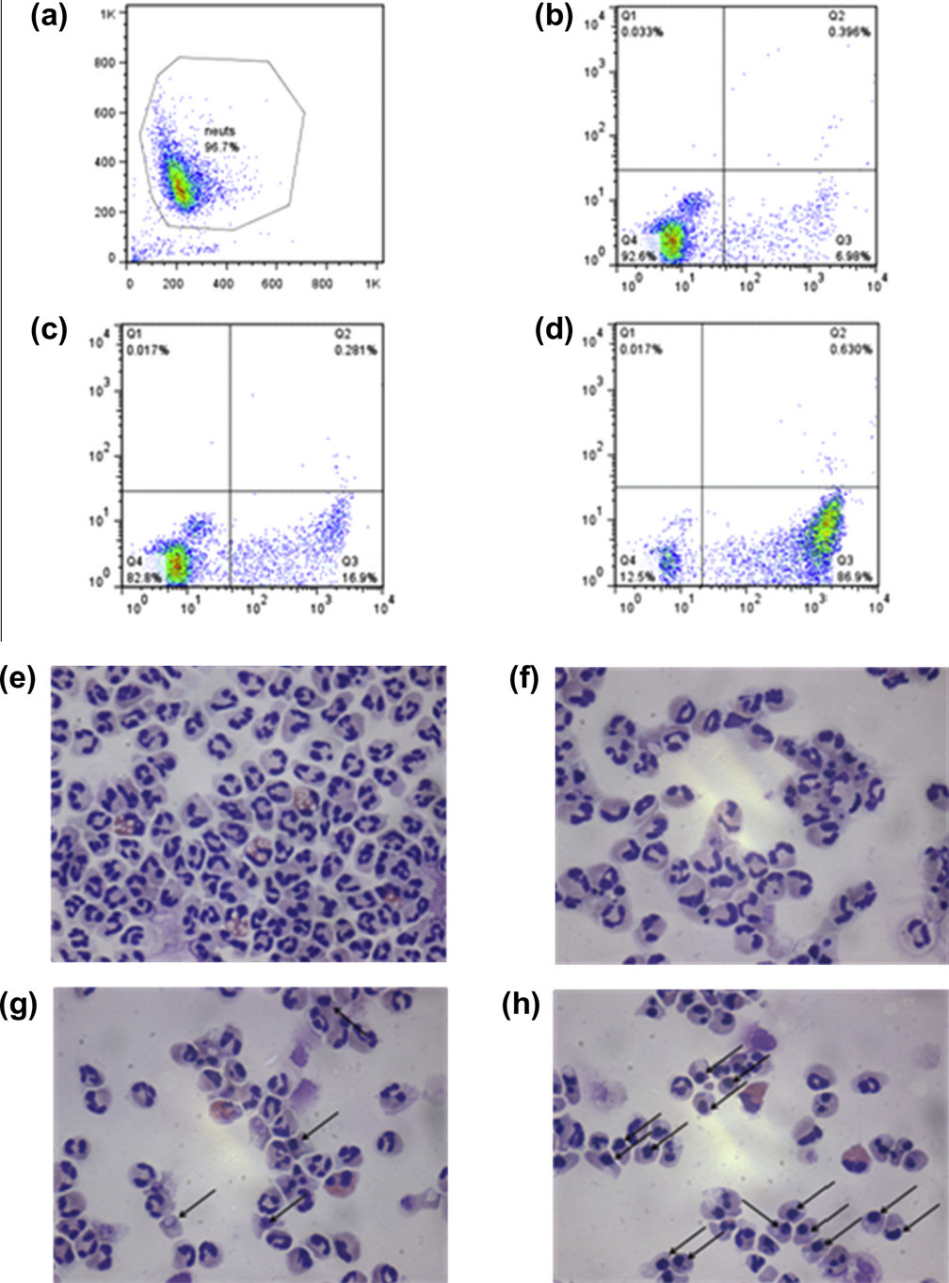
Induction of neutrophil apoptosis by *R*-roscovitine and NO-donor *R*-roscovitine compounds. Representative flow cytometric plot showing forward and side scatter with neutrophils gated (a), and representative flow cytometric plots of annexin V (*x* axis)/propidium iodide (*y* axis) staining after 6 h in culture for control untreated neutrophils (b), neutrophils cultured with *R*-roscovitine 3 μM (c) and neutrophils cultured with compound **9a** 3 μM (d). For annexin V/PI plots lower left quadrant contains viable cells (annexin V negative, PI negative), lower right quadrant contains apoptotic cells (annexin V positive, PI negative), upper right quadrant contains necrotic cells (PI positive). Neutrophil morphology. Representative cyto-centrifuge preparations after staining with Diff-Quick™ (400× light microscopy) in freshly isolated neutrophils (e), untreated neutrophils aged for 6 h (f), neutrophils treated with *R*-roscovitine 3 μM for 6 h (g) and neutrophils treated with compound **9a** 3 μM for 6 h (h). Black arrows indicate apoptotic cells, recognizable by cellular shrinkage and nuclear pyknosis. Contaminating eosinophils (<4%) are also visible, identified by the pink/red staining of their cytoplasm.

**Figure 2:**
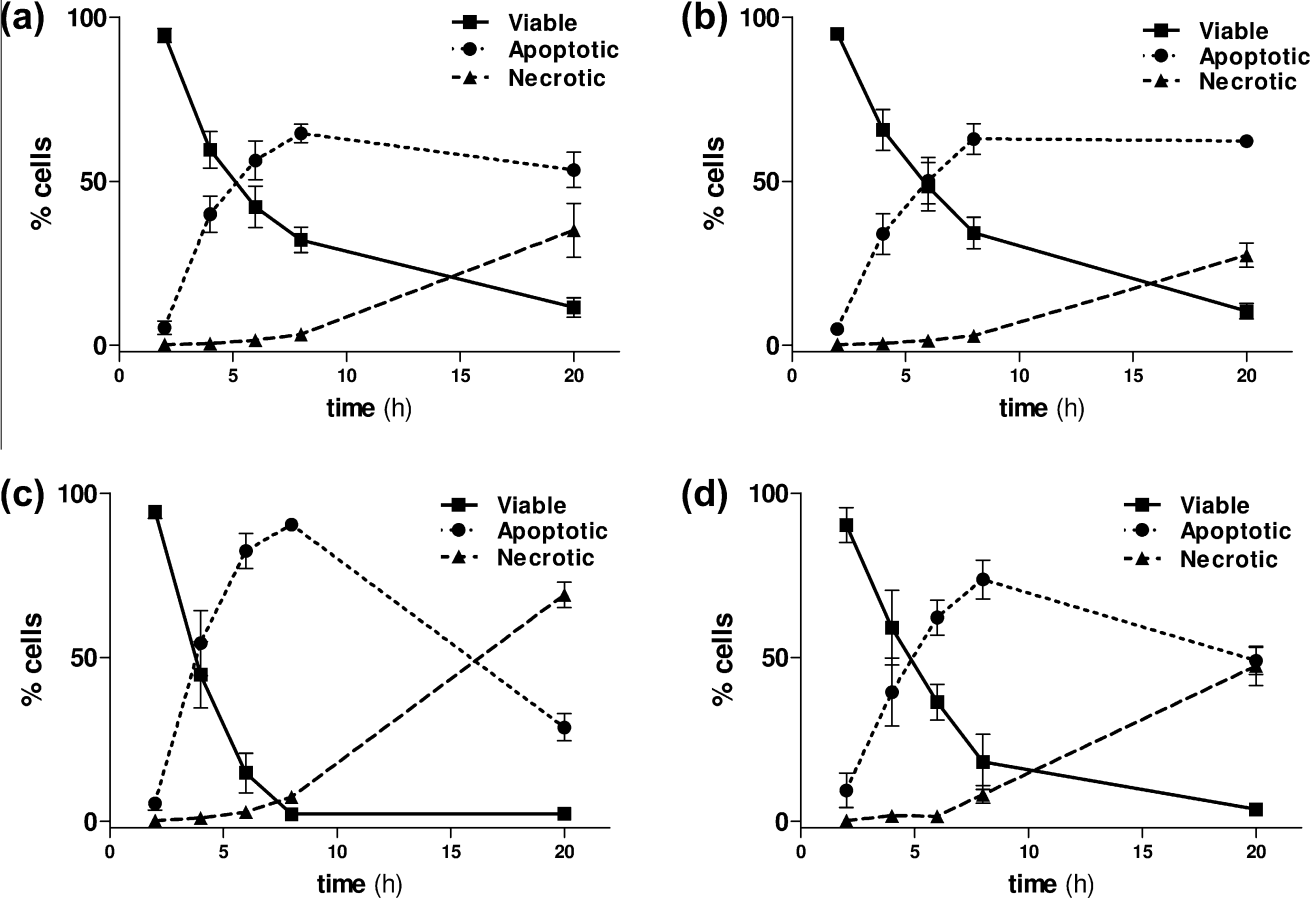
*R*-Roscovitine and NO-donor *R*-roscovitine compounds induce neutrophil apoptosis in a time-dependent manner. Time course demonstrating the percentage (%) of viable (solid line, ■), apoptotic (dotted line, ●) and necrotic (dashed line, ▴) neutrophils induced by treatment with 10 μM *R*-roscovitine (**1**) (panel a), compounds **2** (panel b), **9a** (panel c) and **9c** (panel d). Results expressed as mean ± SEM, *n* = 3.

All the NO-donor *R*-roscovitine derivatives, and the related *des*-NO-reference compounds, were tested for their ability to induce apoptosis in neutrophils isolated from peripheral blood of healthy volunteers. After exposure to the compounds, neutrophil apoptosis was primarily assessed by flow cytometric analysis of fluorescein isothiocyanate (FITC)-labeled annexin V and propidium iodide (PI). This allows discrimination between viable (annexin V and PI −ve), apoptotic (annexin V +ve, PI −ve) and necrotic (annexin V and PI +ve) neutrophils. Representative flow cytometry plots are shown in Figure 1 for *R*-roscovitine and compound **9a** after 6 h of culture. All the results obtained by flow cytometry were also confirmed by light microscopy, with apoptotic neutrophils demonstrating cellular shrinkage and nuclear pyknosis (Fig. 1e–h). Time-course experiments of untreated neutrophils showed that at 6 h the constitutive rate of apoptosis was low (16.8 ± 1.4%). Time-course experiments of neutrophils treated with the lead and the selected compounds **2**, **9a** and **9c** highlighted that all the newly synthesized compounds maintained a *R*-roscovitine-like capability to induce apoptosis over this time period (Fig. 2). Moreover, the same experiments ascertained that secondary as opposed to primary necrosis occurred after 8 h (Fig. 2). This appearance of secondary necrosis at late time points is due to the absence of phagocytes, such as macrophages, being present in the highly pure population of neutrophils used in our experiments. This is well described in the literature^24^ with the temporal distribution of apoptosis occurring prior to the onset of necrosis confirming this as secondary necrosis.

**Figure 3:**
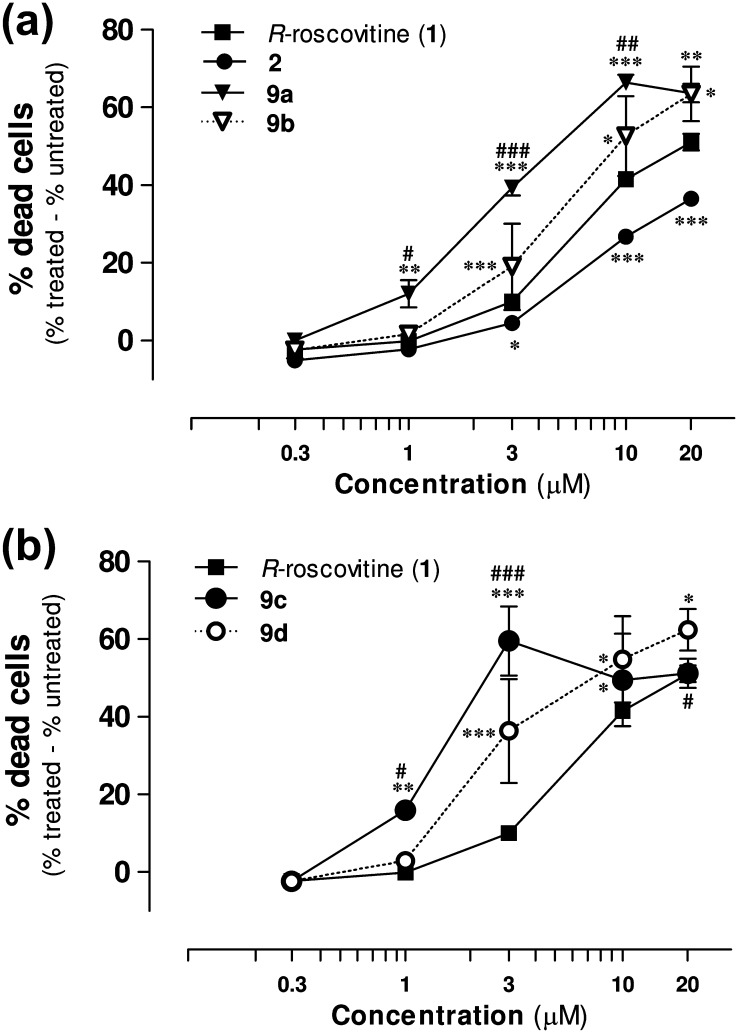
*R*-Roscovitine and NO-donor *R*-roscovitine compounds induce neutrophil apoptosis in a concentration-dependent manner. Percentage of neutrophil death induced by treatment with *R*-roscovitine (**1**), compounds **2** and **9a**,**b** (panel a) and compounds **9c**,**d** (panel b), normalized with respect to the control cells after 6 h of culture. All non-viable, annexin V positive neutrophils are included as at 6 h the percentage of necrotic cells are negligible (Fig. 2). Results expressed as mean ± SEM, *n* = 6–35, analysed by one-way ANOVA with Newman–Keuls *post hoc* test with. ^∗^*p* <0.05, ^∗∗^*p* <0.01,^∗∗∗^*p* <0.001 with respect to *R*-roscovitine and ^#^*p* <0.05, ^##^*p* <0.01, ^###^*p* <0.001 with respect to the corresponding *des*-NO derivative.

**Figure 4:**
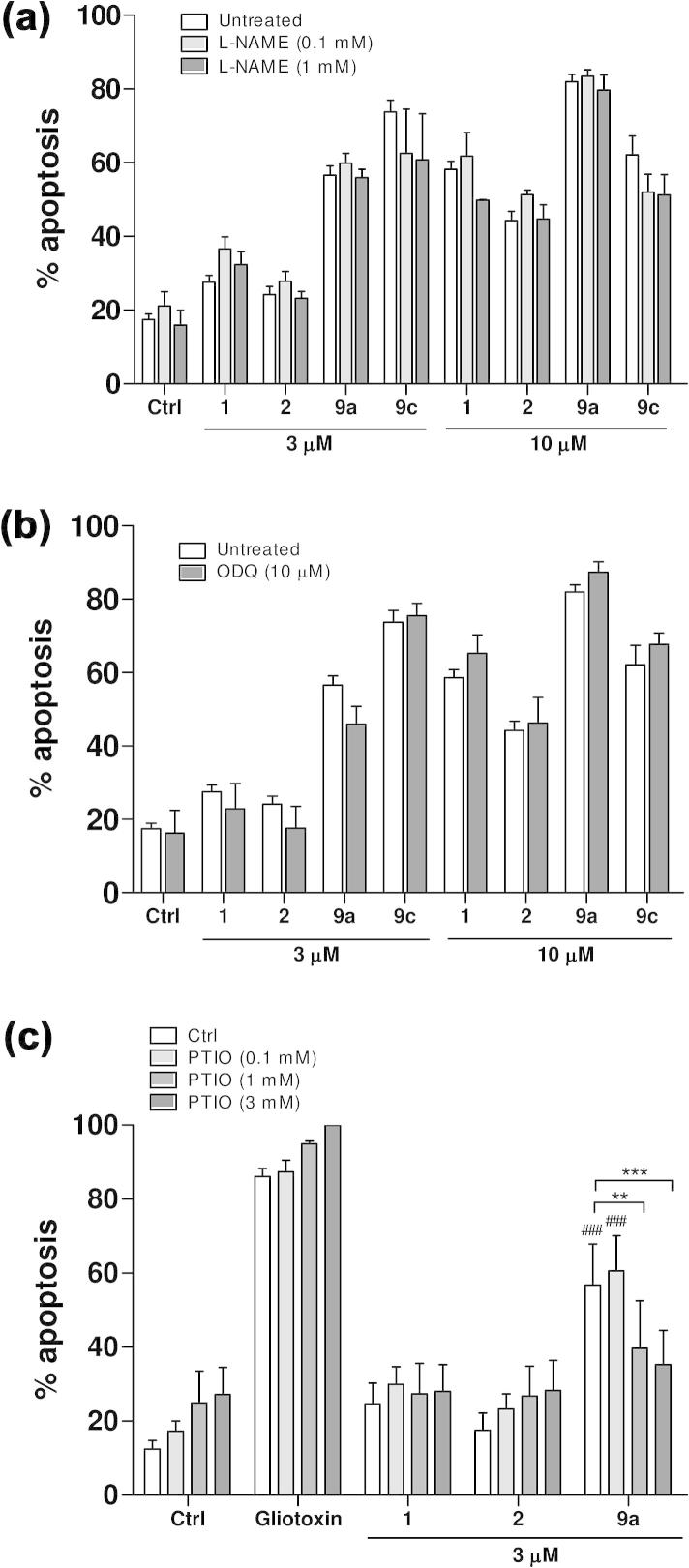
(a and b) Neutrophil apoptosis induced by NO-donor *R*-roscovitine compounds is not affected by inhibition of NO-synthase nor soluble guanylate cyclase. Percentage of neutrophil apoptosis after 6 h of culture using different concentrations of l-NAME (panel a) and 10 μM ODQ (panel b), in the presence of *R*-roscovitine (**1**) and compound **2**, **9a** and **9c** (3 μM and 10 μM). Results expressed as mean ± SEM, *n* = 3. (c) PTIO and NO scavenging. Percentage of neutrophil apoptosis after 6 h of culture using different concentrations of PTIO, in the presence of gliotoxin (1 μg/mL), *R*-roscovitine (**1**), compound **2** and **9a** (3 μM). Results as mean ± SEM, *n* = 5–6, analysed by one-way ANOVA with Newman–Keuls *post hoc* test ^∗^*p* <0.05, ^∗∗^*p* <0.01, ^∗∗∗^*p* <0.001, and ^#^*p* <0.05, ^##^*p* <0.01, ^###^*p* <0.001 with respect to *R*-roscovitine in the same conditions.

Analysis of the concentration–response profiles show that all the new synthesized compounds **2**, **9a**–**d** enhanced neutrophil death by inducing apoptosis in a concentration-dependent manner (Fig. 3), in the range 0.3–20 μM. Both compounds **9a** and **9b** were significantly more potent than the lead. Moreover, the organic nitrate **9a** was markedly more active than its *des*-NO model **9b** in the 1–10 μM range, suggesting an involvement of NO in its additional pro-apoptotic action. Also the furoxan hybrid compounds **9c** and its furazan analogue **9d** show a better dose–response profile with respect to *R*-roscovitine, and, in addition, **9c** is significantly more active than its *des*-NO furazan reference. The pro-drug **2** also enhanced neutrophil death by inducing apoptosis in a concentration-dependent manner, but it was a slightly less potent than *R*-roscovitine. This behavior could be explained by the possibility that the product, over the time of the experiments, acts largely as an intact drug, which should be less active than the lead, in spite of the presence of NO-donor moiety, following the masking of the OH group in the lateral chain. Previous studies with other NO-donor compounds (SIN-1, GEA-3162) showed that NO-donor triggered neutrophil apoptosis is cGMP-independent and involves the concurrent generation of superoxide anion ${\text{O}}_{2}^{-\cdot }$ that could rapidly react with NO to form the powerful oxidizing agent peroxynitrite (ONOO^−^).^19^ Nevertheless, it is not possible to exclude a partial involvement of NOS and of the sGC/cGMP pathway in the regulation of neutrophil apoptosis. Herein, two inhibitors were used with *R*-roscovitine (**1**) and compounds **2**, **9a** and **9c** to examine the role of these NO-related pathways in the induction of apoptosis. Different concentration of *N*-nitro-l-arginine methyl ester (l-NAME), an inhibitor of NO-synthase (NOS), did not modify the behavior of *R*-roscovitine, compounds **2**, **9a** or **9c**, suggesting that their pro-apoptotic effect is not related to iNOS expression or regulation (Fig. 4a). Similarly, the prior administration of ODQ (10 μM), a well known inhibitor of soluble guanylate cyclase (sGC), did not affect the induction of apoptosis (Fig. 4b), excluding involvement of sGC, and confirmed the findings previously reported for other classes of NO-donors. To further investigate the dependency upon NO release for the increased apoptosis observed with the NO-donor containing compounds we co-incubated neutrophils with PTIO, a NO scavenger (Fig. 4c). As expected the presence of PTIO did not affect constitutive neutrophil apoptosis, gliotoxin-induced apoptosis or *R*-roscovitine-induced apoptosis. However PTIO caused a concentration-dependent inhibition of apoptosis when co-incubated with compound **9a**. The rate of apoptosis was not different (*p* >0.05) between *R*-roscovitine and compound **9a** in the presence of >1 mM PTIO, suggesting that the additional apoptosis induced by compound **9a** over *R*-roscovitine was dependent upon NO.

## 3 Conclusion

Basing on the known pro-resolution activity of *R*-roscovitine, novel NO-donor *R*-roscovitine multi-target compounds were prepared with a good yield. The biological results indicate that the new NO-donor *R*-roscovitine compounds described in the present work are endowed with an enhanced roscovitine-like capacity of inducing neutrophil apoptosis, likely involving inhibition of CDKs as well as release of NO. In particular, derivatives **9a** and **9c** were found to be significantly more biologically active than the lead, an effect reduced in their *des*-NO donor derivatives **9b** and **9d**, or by co-incubating **9a** and **9c** with an NO scavenger. This suggests a crucial involvement of the release of NO from the NO-mimetic compounds in their enhanced biological activity.

## 4 Experimental

### 4.1 Reagents and general methods

All the compounds were routinely checked by ^1^H and ^13^C NMR (Bruker Avance 300) at 300 and 75 MHz, respectively, and mass spectrometry (Finnigan-Mat TSQ-700). The following abbreviations are used to indicate the peak multiplicity: s = singlet, d = doublet, t = triplet, m = multiplet. Fx = furoxan ring. Fz = furazan ring. Melting points of unpublished solid derivatives were measured with a capillary apparatus (Buchi B-540). Flash column chromatography was performed on silica gel (Merck Kieselgel 60, 230–400 mesh ASTM) using the reported eluents. Thin layer chromatography (TLC) was carried out on 5 cm × 20 cm plates (Fluka) with a 0.2 mm layer thickness. Purity of final compounds was ⩾95% as detected by RP-HPLC. RP-HPLC analyses were performed on a HP1100 chromatograph system (Agilent Technologies, Palo Alto, CA, USA) on a Nucleosil 100-5C18 Nautilus column (250 × 4.6 mm, 5 μm, Macherey–Nagel), eluted with CH_3_CN/H_2_O + 0.1% TFA 1/1 v/v as mobile phase. Compounds were dissolved in the mobile phase and eluted at flow rates of 1.0 mL min^−1^; the column effluent was monitored at 210, 226, 254 nm referenced against 360 nm. Analysis (C, H, N) of the target compounds was performed by REDOX (Monza). Iscove’s modified Dulbecco’s Modified Eagle’s medium (IMDM), PBS without Ca^2+^/Mg^2+^ and Hank’s balanced salt solution (HBSS) were obtained from PAA. Sterile water and saline were from Baxter. Bovine Serum Albumine (BSA), sterile DMSO, sodium citrate tribasic dihydrate, PBS 10X, N_ω_-nitro-l-arginine methyl ester hydrochloride (l-NAME), and propidium iodide (PI) were purchased from Sigma. Dextran 500 and Percoll were from GE Helthcare. 1*H*-[1,2,4]-oxadiazolo-[4,3-*a*]-quinoxalin-1-one (ODQ) was from Tocris Bioscience. Fluorescein isothiocyanate (FITC) labeled Annexin V was from Roche. 2-Phenyl-4,4,5,5-tetramethylimidazoline-1-oxyl-3-oxide (PTIO) was from Enzo Life Sciences.

### 4.2 Chemistry

#### 4.2.1 (2*R*)-2-(1-Hydroxybut-2-ylamino)-6-benzylamino-9*H*-isopropylpurine (**1**)

*R*-Roscovitine (**1**) was synthesized as reported in literature.25a MS/CI (isobutane): [M+1]^+^ 355. ^1^H NMR (CDCl_3_): *δ*, 7.37–7.23 (m, 6H, *H*_8_ and *H*_Ar_), 6.35 (s br, 1H, N*H*(6)), 5.16 (s br, 1H, CH_2_O*H*), 4.94 (d br, 1H, CHN*H*(2)), 4.76 (s, 2H, C*H*_2_Ph), 4.64–4.50 (m, 1H, C*H*(CH_3_)_2_), 3.91–3.87 (m, 1H, C*H*NH), 3.82–3.78 (m, 1H, C*H*_2_OH), 3.65–3.59 (m, 1H, C*H*_2_OH), 1.72–1.49 (m, 8H, C*H*_2_CH_3_ and CH(C*H*_3_)_2_), 1.00 (t, 3H, CH_2_C*H*_3_). ^13^C NMR (CDCl_3_): *δ*, 160.0, 154.9, 152, 138.9, 134.6, 128.5, 127.7, 127.3, 114.7, 68.2, 56.2, 46.4, 44.4, 25.0, 22.6, 22.5, 10.9. Spectral data are in agreement with those reported in literature.25b

#### 4.2.2 (2*R*)-2-[[6-Benzylamino-9-isopropyl-9*H*-purin-2-yl]amino]butyl 4-(nitrooxy)-butanoate (**2**)

Compound **1** (80 mg, 0.225 mmol) was solubilized in dry pyridine (10 mL). 4-Nitrooxybutirric acid^26^ (74 mg, 0.495 mmol), EDC (52 mg, 0.27 mmol) and DMAP (cat.) were added and the mixture was stirred at room temperature for 4 h. The mixture was treated with HCl 1 N, extracted with CH_2_Cl_2_ (3 × 15 mL) and the combined organic phases were washed with HCl 1 N (3 × 10 mL), brine (20 mL), dried (Na_2_SO_4_), filtered and evaporated in vacuo. The crude residue was purified by flash cromatography eluting with CH_2_Cl_2_ gradient to CH_2_Cl_2_/MeOH 1% to obtain the pure product as a yellow oil (50 mg, yield 48%). MS/CI (isobutane): [M+1]^+^ 486. ^1^H NMR (CDCl_3_): *δ*, 7.36 (s, 1H, *H*_8_), 7.33–7.21 (m, 5H, H_Ar_), 6.47 (s br, 1H, N*H*(6)), 4.80–4.77 (m, 3H, CHN*H*(2) and C*H*_2_Ph), 4.65–4.55 (m, 1H, C*H*(CH_3_)_2_), 4.44 (t, 2H, C*H*_2_ONO_2_), 4.24–4.16 (m, 3H, C*H*_2_O(CO) and C*H*NH), 2.41 (t, 2H, C*H*_2_(CO)O), 2.04–1.97 (m, 2H, CH_2_C*H*_2_CH_2_), 1.68–1.49 (m, 8H, C*H*_2_CH_3_ and CH(C*H*_3_)_2_), 0.97 (t, 3H, CH_2_C*H*_3_). ^13^C NMR (CDCl_3_): *δ*, 172.3, 160.0, 154.9, 151.0, 139.4, 134.6, 128.5, 127.6, 127.1, 114.6, 72.0, 66.2, 51.5, 46.2, 44.3, 30.1, 24.9, 22.58, 22.57, 22.2, 10.6. HPLC purity (CH_3_CN/H_2_O + 0.1% TFA, 1/1): 99%. Elemental analysis for C_23_H_31_N_7_O_5_. Calculated: C, 56.90; H, 6.44; N, 20.19. Found: C, 57.49; H, 6.46; N, 19.99.

#### 4.2.3 6-Benzylthio-2-iodo-9*H*-purine (**5**)

9-(2′,3′,5′-Tri-*O*-acetyl-β-d-ribofuranosyl)-6-chloro-2-iodopurine **3**^27^ (4.42 g, 8.20 mmol) was suspended in water (25 mL) and EtOH (50 mL), then H_2_SO_4_ conc was added (2.5 mL) and the mixture was refluxed for 3 h. The mixture was concentrated in vacuo to a volume of approximatively 30 mL and NaOH 10% was added until the pH reached 4.5. The aqueous phase was extracted with CH_2_Cl_2_ (4 × 50 mL), Et_2_O (3 × 50 ml), dried (Na_2_SO_4_), filtered and evaporated in vacuo to obtain a crude yellow solid. The crude was purified by flash cromatography eluting with CH_2_Cl_2_ gradient to CH_2_Cl_2_/MeOH 5% to obtain 9-*H*-6-chloro-2-iodopurine (**4**) pure enough as an intermediate (960 mg). MS/CI (isobutane): [M+1]^+^ 281, 283. Compound **4** (3.42 mmol) was dissolved in EtOH (80 mL). DIPEA (1.17 mL, 6.84 mmol) and benzylmercaptane (804 μL, 6.84 mmol) were added and the mixture was refluxed under nitrogen atmosphere for 4 h. The solvent was evaporated in vacuo, treated with water (30 mL), extracted with AcOEt (3 × 50 mL) and the combined organic phases were washed with brine (40 mL), dried (Na_2_SO_4_), filtered and evaporated under reduced pressure. The crude product was treated with hexane and collected on a buchner funnel to obtain the desired product (960 mg) as a yellow solid (overall yield 31%). An analytical sample was obtained by flash cromatography eluting with CH_2_Cl_2_/MeOH 2% to obtain the title product as a white solid. MS/CI (isobutane): [M+1]^+^ 369. ^1^H NMR (DMSO-*d*_6_): *δ*, 13.63 (s br, 1H, N*H*), 8.40 (s, 1H, *H*_8_), 7.51 (d, 2H, H_Ar 2′,6′_), 7.44–7.23 (m, 3H, H_Ar 3′,4′,5′_), 4.57 (s, 2H, C*H*_2_Ph). ^1^H NMR spectrum is in keeping with that reported in literature.^28^

#### 4.2.4 6-Benzythio-2-iodo-9-isopropyl-9*H*-purine (**6**)

Compound **5** (900 mg, 2.44 mmol) was solubilized in DMSO (20 mL), K_2_CO_3_ (1.01 g, 7.32 mmol) and 2-bromopropane (574 μL, 6.1 mmol) were added and the mixture was stirred at 50 °C overnight. The mixture was treated with water (50 mL), extracted with AcOEt (3 × 60 mL), dried (Na_2_SO_4_), filtered and evaporated in vacuo to obtain a yellow oil. The crude product was purified by flash cromatography eluting with PE/CH_2_Cl_2_ 1:1 gradient to CH_2_Cl_2_ to obtain the desired product as a white solid (500 mg, 50% yield). MS/CI (isobutane): [M+1]^+^ 411. ^1^H NMR (CDCl_3_): *δ*, 7.93 (s, 1H, *H*_8_), 7.51 (d, 2H, H_Ar 2′,6′_), 7.34–7.21 (m, 3H, H_Ar 3′,4′,5′_), 4.90–4.81 (m, 1H, C*H*(CH_3_)_2_), 4.56 (s, 2H, C*H*_2_Ph), 1.61–1.57 (m, 6H, CH(C*H*_3_)_2_). ^13^C NMR (CDCl_3_): *δ*, 161.5, 148.9, 140.0, 137.2, 131.1, 129.5, 128.4, 127.4, 118.2, 47.5, 33.3, 22.7. ^1^H NMR and ^13^C NMR are identical to the spectra reported in literature.^28^

#### 4.2.5 (2*R*)-2-[[6-Benzylthio-9-isopropyl-9*H*-purin-2-yl]amino]-butanol (**7**)

Compound **6** (0.5 g, 1.22 mmol) was suspended in *n*-butanol (20 mL). (*R*)-2-Amino-1-butanol was added (434 mg, 4.88 mmol) and the reaction was refluxed for 5 days. The solvent was evaporated in vacuo using an oil pump and the crude residue was purified by flash cromatography eluting with CH_2_Cl_2_ gradient to CH_2_Cl_2_/MeOH 1.5% to obtain pure product as a colourless oil (340 mg, yield 75%). MS/CI (isobutane): [M+1]^+^ 372. ^1^H NMR (CDCl_3_): *δ*, 7.67 (s, 1H, H_8_), 7.43 (d, 2H, H_Ar 2′,6′_), 7.29–7.26 (m, 3H, H_Ar 3′,4′,5′_), 5.20 (d br, 1H, CH_2_O*H*), 4.70–4.63 (m, 1H, C*H*(CH_3_)_2_), 4.56 (s, 2H, C*H*_2_Ph), 4.02–3.90 (m, 1H, C*H*NH), 3.81 (m, 1H, C*H*_2_OH), 3.69 (m, 1H, C*H*_2_OH), 1.80–1.53 (m, 8H, C*H*_2_CH_3_ and CH(C*H*_3_)_2_), 1.02 (t, 3H, CH_2_C*H*_3_). ^13^C NMR (CDCl_3_): *δ*, 160.9, 158.7, 149.8, 137.6, 137.4, 129.0, 128.4, 127.1, 125.5, 66.8, 55.9, 46.8, 32.7, 24.80, 22.45, 22.39, 10.8.

#### 4.2.6 (2*R*)-2-[[6-Benzylsulfonyl-9-isopropyl-9*H*-purin-2-yl]amino]-butanol (**8**)

Derivative **7** (170 mg, 0.45 mmol) was solubilized in CH_2_Cl_2_ (10 mL), the mixture was cooled at 0 °C and *m*-CPBA was added (340 mg, 1.35 mmol). The mixture was stirred at room temperature for 3 h, then diluted with water. The two layers were divided and the water phase was extracted with fresh CH_2_Cl_2_ (15 mL). The combined organic phases were washed with NaHCO_3_ 5% (2 × 10 mL), water (10 mL), brine (10 mL), dried (Na_2_SO_4_), filtered and evaporated in vacuo. The crude product was purified eluting with CH_2_Cl_2_ gradient to CH_2_Cl_2_/MeOH 2% to obtain the title product as a yellow oil (82 mg, yield 45%). Derivative **8** was stored at −18 °C or immediately reacted to avoid a possible decomposition. MS/CI (isobutane): [M+1]^+^ 404. ^1^H NMR (CDCl_3_): *δ*, 7.96 (s, 1H, *H*_8_), 7.74–7.26 (m, 5H, H_Ar_), 5.95 (d br, 1H, N*H*(2)), 4.84 (s, 2H, C*H*_2_Ph), 4.79–4.68 (m, 1H, C*H*(CH_3_)_2_), 4.04 (s br, 1H, C*H*NH), 3.82–3.80 (m, 1H, C*H*_2_OH), 3.72–3.66 (m, 1H, C*H*_2_OH), 1.76–1.58 (m, 8H, C*H*_2_CH_3_ and CH(C*H*_3_)_2_), 0.99 (t, 3H, CH_2_C*H*_3_). ^13^C NMR (CDCl_3_): *δ*, 158.5, 156.0, 153.3, 142.3, 131.7, 129.6, 129.0, 126.6, 123.3, 64.2, 59.4, 53.5, 47.4, 24.3, 22.2, 22.0, 10.7.

#### 4.2.7 General procedure for preparation of **9a**–**d**

Compound **8** (107 mg, 0.265 mmol) was solubilized in EtOH (10 mL). Triethylamine (111 μL, 0.795 mmol) and the correct amino derivatives (**12**, **14**, **16** or **18**) (1.5 equiv) were added, and the mixture was stirred at 40 °C for 5 days. The solvent was evaporated in vacuo, dissolved in CH_2_Cl_2_ (30 mL), washed with water (2 × 20 mL), dried (Na_2_SO_4_), filtered and evaporated under reduced pressure. The crude residue was purified by flash cromatography eluting with CH_2_Cl_2_/AcOEt 6:4 gradient to 1:9 to obtain pure products. Derivatives **9a**–**d** were stored at −18 °C.

##### 4.2.7.1 (2*R*)-2-[[9-Isopropyl-6-[[4-[3-(nitrooxy)propoxy]benzyl]amino]-9*H*-purin-2-yl]amino]-butanol (**9a**)

Pale yellow oil (yield 40%). MS/CI (isobutane): [M+1]^+^ 474. ^1^H NMR (CDCl_3_): *δ*, 7.36 (s, 1H, *H*_8_), 7.25 (d, 2H, H_Ar 2′,6′_), 6.79 (d, 2H, H_Ar 3′,5′_), 6.54 (s br, 1H, N*H*(6)), 5.20 (s br, 1H, O*H*), 5.02 (d br, 1H, N*H*(2)), 4.66–4.60 (m, 4H, C*H*_2_Ph and C*H*_2_ONO_2_), 4.58–4.54 (m, 1H, C*H*(CH_3_)_2_), 4.04 (t, 2H, C*H*_2_OPh), 3.92–3.87 (m, 1H, C*H*NH), 3.82–3.77 (m, 1H, C*H*_2_OH), 3.65–3.59 (m, 1H, C*H*_2_OH), 3.21–3.13 (m, 2H, CH_2_C*H*_2_CH_2_), 1.64–1.47 (m, 8H, C*H*_2_CH_3_ and CH(C*H*_3_)_2_), 0.99 (t, 3H, CH_2_C*H*_3_). ^13^C NMR (CDCl_3_): *δ*, 160.0, 157.8, 154.8, 150.2, 134.4, 131.5, 129.0, 114.7, 114.4, 70.0, 67.8, 63.5, 56.1, 46.3, 43.7, 26.9, 24.9, 22.7, 22.5, 10.9. HPLC purity (CH_3_CN/H_2_O + 0.1% TFA, 1/1): 98%. Elemental analysis for C_22_H_31_N_7_O_5_·1/2H_2_O. Calculated: C, 54.76; H, 6.68; N, 20.32. Found: C, 55.14; H, 6.53; N, 20.10.

##### 4.2.7.2 (2*R*)-2-[[9-Isopropyl-6-[[4-[3-(methoxy)propoxy]benzyl]amino]-9*H*-purin-2-yl]amino]-butanol (**9b**)

Pale yellow oil (yield 42%). MS/CI (isobutane): [M+1]^+^ 443. ^1^H NMR (CDCl_3_): *δ*, 7.23–7.20 (m, 4H, *H*_8_, H_Ar 2′,6′_ and N*H*(6)), 6.78 (d, 2H, H_Ar 3′,5′_), 5.80 (s br, 1H, O*H*), 5.26 (d br, 1H, N*H*(2)), 4.64 (s br, 2H, C*H*_2_Ph), 4.56–4.47 (m, 1H, C*H*(CH_3_)_2_), 4.00 (t, 2H, C*H*_2_OPh), 3.93–3.91 (m, 1H, C*H*NH), 3.77–3.73 (m, 1H, C*H*_2_OH), 3.64–3.61 (m, 1H, C*H*_2_OH), 3.51 (t, 2H, C*H*_2_OCH_3_), 3.31 (s, 3H, OC*H*_3_), 2.04–1.96 (m, 2H, CH_2_C*H*_2_CH_2_), 1.62–1.41 (m, 8H, C*H*_2_CH_3_ and CH(C*H*_3_)_2_), 0.96 (t, 3H, CH_2_C*H*_3_). ^13^C NMR (CDCl_3_): *δ*, 159.9, 158.1, 154.8, 150.3, 134.3, 131.2, 129.2, 114.7, 113.9, 69.2, 66.5, 64.7, 58.6, 55.6, 46.2, 43.6, 29.5, 24.7, 22.6, 22.3, 10.9. HPLC purity (CH_3_CN/H_2_O + 0.1% TFA, 1/1): 99%. Elemental analysis for C_23_H_34_N_6_O_3_. Calculated: C, 62.42; H, 7.74; N, 18.99. Found: C, 62.48; H, 7.78; N, 18.85.

##### 4.2.7.3 4-[[4-[[2-[[(1*R*)-1-(Hydroxymethyl)propyl]amino]-9-isopropyl-9*H*-purin-6-yl]amino]-benzyl]phenoxy]methyl]furoxan-3-carboxamide (**9c**)

Yellow solid (yield 50%). MS/CI (isobutane): [M+1]^+^ 512. Mp 136 °C dec. ^1^H NMR (CD_3_OD): *δ*, 7.76 (s, 1H, *H*_8_), 7.31 (d, 2H, H_Ar 3′,5′_), 6.98 (d, 2H, H_Ar 2′,6′_), 5.38 (s, 2H, Fx-C*H*_2_OPh), 4.67–4.60 (m, 3H, C*H*_2_Ph and C*H*(CH_3_)_2_), 3.94 (m, 1H, C*H*NH), 3.61 (m, 2H, C*H*_2_OH), 1.70–1.52 (m, 8H, C*H*_2_CH_3_ and CH(C*H*_3_)_2_), 0.96 (t, 3H, CH_2_C*H*_3_). ^13^C NMR (CD_3_OD): *δ*, 161.2, 158.6, 158.1, 156.6, 156.0, 152.1, 136.4, 134.3, 130.0, 116.1, 114.6, 111.8, 65.5, 62.7, 55.9, 47.9, 44.5, 25.5, 22.7, 22.6, 11.1. HPLC purity (CH_3_CN/H_2_O + 0.1% TFA, 1/1): 95%. Elemental analysis for C_23_H_29_N_9_O_5_·1/4H_2_O. Calculated: C, 53.53; H, 5.76; N, 24.42. Found: C, 53.65; H, 5.72; N, 24.10.

##### 4.2.7.4 4-[[4-[[2-[[(1*R*)-1-(Hydroxymethyl)propyl]amino]-9-isopropyl-9*H*-purin-6-yl]amino]-benzyl]phenoxy]methyl]furazan-3-carboxamide (**9d**)

White foam (yield 49%). MS/CI (isobutane): [M+1]^+^ 496. ^1^H NMR (CDCl_3_ + CD_3_OD): *δ*, 7.67 (s, 1H, *H*_8_), 7.30 (d, 2H, H_Ar 3′,5′_), 6.94 (d, 2H, H_Ar 2′,6′_), 5.45 (s, 2H, Fz-C*H*_2_OPh), 4.72–4.56 (m, 3H, C*H*_2_Ph and C*H*(CH_3_)_2_), 4.00–3.93 (m, 1H, C*H*NH), 3.67–3.65 (m, 2H, C*H*_2_OH), 1.73–1.51 (m, 8H, C*H*_2_CH_3_ and CH(C*H*_3_)_2_), 0.97 (t, 3H, CH_2_C*H*_3_). ^13^C NMR (CD_3_OD): *δ*, 160.6, 160.3, 157.9, 155.5, 153.0, 151.2, 149.1, 135.7, 133.5, 129.6, 115.6, 114.3, 65.6, 60.5, 55.6, 47.3, 44.2, 25.2, 22.6, 22.5, 11.0. HPLC purity (CH_3_CN/H_2_O + 0.1% TFA, 1/1): 95%. Elemental analysis for C_23_H_29_N_9_O_4_·1/4H_2_O. Calculated: C, 55.24; H, 5.95; N, 25.21. Found: C, 55.52; H, 5.97; N, 25.46.

#### 4.2.8 3-[4-(Aminomethyl)phenoxy]propyl nitrate (**12**)

*tert*-Butyl-*N*-(4-hydroxybenzyl)carbamate **10**^29^ (730 mg, 3.27 mmol) was solubilized in CH_3_CN (20 mL), K_2_CO_3_ (565 mg, 4.09 mmol), 3-nitrooxypropyl bromide (**11**)^30^ (0.5 g, 2.73 mmol), KI (cat.) and DMF (0.5 mL) were added. The mixture was stirred at 50 °C for 20 h, evaporated under reduced pressure, treated with water (20 mL), extracted with CH_2_Cl_2_ (3 × 30 ml) and the combined organic phases were washed with brine (35 mL), dried (Na_2_SO_4_), filtered and evaporated. The crude product was purified by flash cromatography eluting with PE/*i*PrOH 1% to obtain *tert*-butyl-(4-(3-nitrooxy)propoxybenzyl)carbamate (510 mg). This product (1.56 mmol) was then solubilized in CH_2_Cl_2_ (15 mL). Trifluoroacetic acid was added (1.5 mL) and the mixture was stirred at room temperature for 3 h. The solvent was evaporated in vacuo, cooled at 0 °C and NaHCO_3_ 10% was added. The mixture was stirred for 10 min, extracted with CH_2_Cl_2_ (3 × 20 ml) and the combined organic phases were washed with water (2 × 15 mL), brine (20 mL), dried (Na_2_SO_4_), filtered and evaporated. The crude residue was purified by flash cromatography eluting with CH_2_Cl_2_ gradient to CH_2_Cl_2_/MeOH 5% to obtain the pure product as a colourless oil (160 mg, overall yield 30%). MS/CI (isobutane): [M+1]^+^ 227. ^1^H NMR (CD_3_OD): *δ*, 7.23 (d, 2H, *H*_Ar 3′,5′_), 6.84 (d, 2H, *H*_Ar 2′,6′_), 4.66 (t, 2H, CH_2_ONO_2_), 4.04 (t, 2H, C*H*_2_OPh), 3.79 (s, 2H, PhC*H*_2_), 2.24–2.15 (m, 2H, CH_2_C*H*_2_CH_2_). ^13^C NMR (CD_3_OD): *δ*, 157.5, 135.2, 128.5, 114.5, 70, 63.6, 45.8, 27. This intermediate was not further characterized but reacted immediately.

#### 4.2.9 4-(3-Methoxypropoxy)benzylamine (**14**)

Compound **10** (914 mg, 4.09 mmol) was solubilized in dry THF (30 mL). *t*BuO^−^K^+^ (460 mg, 4.09 mmol) was added and the mixture was stirred at room temperature for 10 min. 3-Methoxypropyl tosylate (**13**)^31^ (1.0 g, 4.09 mmol) was solubilized in dry THF (5 mL) and added to the mixture that was refluxed for 4 h. The solvent was evaporated under reduced pressure, and the residue was dissolved with water (40 mL), extracted with CH_2_Cl_2_ (3 × 30 mL), and the combined organic phases were washed with brine (45 mL), dried (Na_2_SO_4_), filtered and evaporated in vacuo. The crude product was purified by flash cromatography eluting with PE/*i*PrOH 4% to obtain *tert*-butyl-(4-(3-methoxy)propoxybenzyl)carbamate (1.10 g), enough pure to be used in the next reaction step. The product (3.72 mmol) was solubilized in CH_2_Cl_2_ (40 mL). Trifluoroacetic acid was added (4 mL) and the mixture was stirred at room temperature for 3 h. The solvent was evaporated in vacuo, and NaOH 10% was added. The aqueous phase was extracted with CH_2_Cl_2_ (3 × 20 mL) and the combined organic phases were washed with water (2 × 15 mL), brine (20 mL), dried (Na_2_SO_4_), filtered and evaporated. The crude residue was purified by flash cromatography eluting with CH_2_Cl_2_ gradient to CH_2_Cl_2_/MeOH 5% to obtain the title product as a colourless oil (480 mg, overall yield 60%). ^1^H NMR (CD_3_OD): *δ*, 7.26 (d, 2H, H_Ar 2′,6′_), 6.88 (d, 2H, H_Ar 3′,5′_), 4.03–3.97 (m, 2H, C*H*_2_OPh), 3.79 (s, 2H, PhC*H*_2_), 3.55–3.51 (m, 2H, C*H*_2_OCH_3_), 3.32 (s, 3H, OC*H*_3_), 2.03–1.95 (m, 2H, CH_2_C*H*_2_CH_2_). This intermediate was not further characterized but reacted immediately.

#### 4.2.10 4-[[4-(Aminomethyl)phenoxy]methyl]furoxan-3-carboxamide (**16**)

Compound **10** (500 mg, 2.23 mmol) was solubilized in dry THF (25 mL). CAS 1609 (**15**) (534 mg, 3.34 mmol), produced by Cassella–Hoechst Co., and PPh_3_ (1.17 g, 4.46 mmol) were added. The mixture was cooled at 0 °C, DIAD (877 μL, 4.46 mmol) was solubilized in dry THF (2 mL) and added dropwise. The mixture was stirred at room temperature overnight. The solvent was evaporated under reduced pressure and the residue was solubilized in CH_2_Cl_2_ (20 mL), cooled at 0 °C, added with trifluoroacetic acid (2 mL) and stirred at room temperature for 3 h. The solvent was evaporated and the residue treated with water (45 mL) and extracted with AcOEt (2 × 50 mL). The water phase was added with Na_2_CO_3_ 10% and newly extracted with AcOEt (4 × 40 mL). The combined organic phases were dried (K_2_CO_3_), filtered and evaporated in vacuo. The crude product was purified by flash chromatography eluting with CH_2_Cl_2_/MeOH 5% to obtain pure product as a yellow solid (400 mg, yield 67%). MS/CI (isobutane): [M+1]^+^ 265. Mp 145 °C dec. ^1^H NMR (CD_3_OD): *δ*, 7.29 (d, 2H, H_Ar 3′,5′_), 7.01 (d, 2H, H_Ar 2′,6′_), 5.40 (s, 2H, Fx-C*H*_2_OPh), 3.75 (s, 2H, C*H*_2_Ph). This intermediate was not further characterized but reacted immediately.

#### 4.2.11 4-[(4-(Aminomethyl)phenoxy)methyl]furazan-3-carboxamide (**18**)

Compound **10** (500 mg, 2.23 mmol) was solubilized in dry CH_2_Cl_2_ (50 mL), DBU (490 μL, 3.34 mmol) and 4-(bromomethyl)-1,2,5-oxadiazole-3-carboxamide (**17**) (460 mg, 2.23 mmol) were added. The mixture was stirred at room temperature overnight, cooled at 0 °C, added with trifluoroacetic acid (5 mL) and stirred at room temperature for 1 h. The solvent was evaporated under reduced pressure and the residue treated with water (40 mL) and extracted with AcOEt (2 × 50 mL). The water phase was added with Na_2_CO_3_ 10% and extracted with fresh AcOEt (4 × 40 mL). The combined organic phases were washed with brine (60 mL), dried (Na_2_SO_4_), filtered and evaporated in vacuo. The crude residue was purified by flash cromatography eluting with CH_2_Cl_2_/MeOH 5% to obtain pure product as a pale yellow oil (350 mg, yield 63%). MS/CI (isobutane): [M+1]^+^ 249. ^1^H NMR (CD_3_OD + *d*_6_-DMSO): *δ*, 7.27 (d, 2H, H_Ar 3′,5′_), 7.00 (d, 2H, H_Ar 2′,6′_), 5.49 (s, 2H, Fz-C*H*_2_OPh), 4.58 (s br, 2H, CH_2_N*H*_2_), 3.72 (s, 2H, C*H*_2_Ph). This intermediate was not further characterized but reacted immediately.

### 4.3 Neutrophil culture and assessment of apoptosis

#### 4.3.1 Cells isolation and culture

Neutrophils were isolated from peripheral blood of healthy volunteers as described;^15^ ethics approval was obtained from the Lothian Research Ethics Committee (approval no. 08/S1103/38). Blood (40 mL aliquots) was collected into 4 mL sodium citrate 3.8% (3.8 g in 100 mL sterile water), mixed by gentle inversion and centrifugation (350*g*, 20 min). Platelet rich plasma (PRP) was removed and 6 mL of dextran 6% (6 g in 100 mL 0.9% NaCl) was added, together with pre-warmed sterile saline up to 50 mL. After 30 min sedimentation, the upper layer (containing leukocytes) was separated and washed with saline. Percoll™ (27 mL) was made isotonic with PBS (without cations) 10× (3 mL) and different Percoll™ solutions in PBS (81%, 70% and 55%) were then prepared. A discontinuous gradient was made up, carefully overlayering 3 mL of each Percoll™ solutions and resuspending the leukocyte pellets in the 55% layer. Leukocytes were then divided into the different populations and separated from the residual erythrocytes by centrifugation (720*g*, 20 min): mononuclear cells were retained at the upper gradient boundary (55:70) and granulocytes at the lower boundary (70:81). Granulocytes were collected and washed two times with PBS (without cations).

Neutrophils (>96% purity, with 1–4% contaminating eosinophils) were re-suspended at 5 × 10^6^/mL in IMDM containing penicillin (100 U/mL) and streptomycin (100 μg/mL) and supplemented with 0.1% w/v bovine serum albumin (BSA).^32^ Cells were cultured in flat-bottomed flexible 96-well Falcon polypropylene plates (final volume 150 μL each well) in a 5% CO_2_ atmosphere at 37 °C and treated in duplicate with the corresponding compounds, added immediately prior of the addition of cells. In the experiments with the inhibitors l-NAME and ODQ, the cells were pre-incubated for 15 min in the presence of the inhibitor prior to the addition of the other tested compounds, to avoid a possible interference of endogenous NOS and sGC.^33^

#### 4.3.2 Preparation of the drugs

All tested compounds were prepared in a 50 mM stock solution in sterile DMSO and diluted daily in IMDM as necessary. l-NAME and PTIO solution was prepared directly in IMDM. ODQ was prepared in a NaOH 0.01% 10 mM stock solution prior to the correct dilution in IMDM. The percentage of DMSO in the final cell culture was within 0.04–0.12%.

#### 4.3.3 Assessment of neutrophil apoptosis

Neutrophil apoptosis was primarily assessed by flow cytometry using a FACScan (Becton Dickinson) as previously described.^24^ Annexin V was diluted 1/500 in the binding buffer (2.5 mL of 1 M CaCl_2_ in 500 mL HBSS) and 280 μL added to 20 μL of cells (5 × 10^6^/mL). Samples were then incubated on ice for 10 min protecting from light. Prior to analysis on the flow cytometer, 1 μL of PI (1 mg/mL) per sample was added. Data from the flow cytometry experiments were collected with Cell Quest Software and analysed with Flow Jo (Tree Star Inc., version 7.6). Statistical analysis was performed using Prism 5 (GraphPad Software, Inc., version 5.00). Apoptosis was also confirmed by cyto-centrifuging 80 μL of 5 × 10^6^/mL cells (300 rpm, 3 min), fixing the cells on the slide for 2 min in methanol and staining with Diff-Quick™ (Gamidor). Apoptotic morphology was assessed at 40× and 100× objective on a light microscope using standard criteria.
